# Preclinical models of head and neck squamous cell carcinoma for a basic understanding of cancer biology and its translation into efficient therapies

**DOI:** 10.1186/s41199-020-00056-4

**Published:** 2020-07-23

**Authors:** Ingeborg Tinhofer, Diana Braunholz, Konrad Klinghammer

**Affiliations:** 1 Charité – Universitätsmedizin Berlin, corporate member of Freie Universität Berlin, Humboldt-Universität zu Berlin, and Berlin Institute of Health, Department of Radiooncology and Radiotherapy, Berlin, Germany; 2 German Cancer Research Center (DKFZ), Heidelberg and German Cancer Consortium (DKTK) Partner Site Berlin, Berlin, Germany; 3 Charité – Universitätsmedizin Berlin, corporate member of Freie Universität Berlin, Humboldt-Universität zu Berlin, and Berlin Institute of Health, Department of Hematology and Oncology, Berlin, Germany

**Keywords:** Preclinical models, Patient-derived xenografts, Patient-derived primary cultures, Organoids, Personalized medicine

## Abstract

Comprehensive molecular characterization of head and neck squamous cell carcinoma (HNSCC) has led to the identification of distinct molecular subgroups with fundamental differences in biological properties and clinical behavior. Despite improvements in tumor classification and increased understanding about the signaling pathways involved in neoplastic transformation and disease progression, current standard-of-care treatment for HNSCC mostly remains to be based on a stage-dependent strategy whereby all patients at the same stage receive the same treatment. Preclinical models that closely resemble molecular HNSCC subgroups that can be exploited for dissecting the biological function of genetic variants and/or altered gene expression will be highly valuable for translating molecular findings into improved clinical care. In the present review, we merge and discuss existing and new information on established cell lines, primary two- and three-dimensional ex vivo tumor cultures from HNSCC patients, and animal models. We review their value in elucidating the basic biology of HNSCC, molecular mechanisms of treatment resistance and their potential for the development of novel molecularly stratified treatment.

## Background

Head and neck cancer is the seventh most common cancer type by incidence and mortality, with 890,000 new cases and 450,000 deaths worldwide in 2018 [1]. Treatment remains challenging with current therapies resulting in five-year survival rates below 50% for patients with locally advanced disease [2]. Drug resistance and toxicity limit the efficacy of chemotherapeutics such as cis- or carboplatin, 5-fluorouracil, and taxanes. The introduction of targeted agents such as cetuximab, nivolumab or pembrolizumab improved the outcome but did not overcome the problem of primary or acquired treatment resistance in the majority of patients [3–5]. Only very few biomarkers are currently used in clinical practice or have actually proceeded towards validation for routine use [6]. Reliable preclinical models are therefore critical to better understand the molecular mechanisms involved in HNSCC treatment resistance and progression, and to develop more effective therapeutic strategies.

**Fig. 1:**
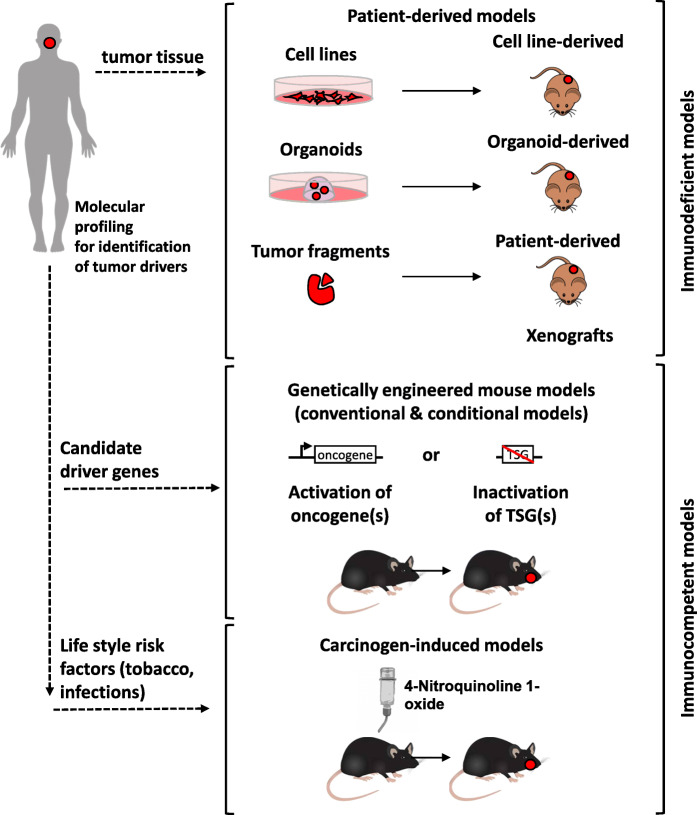
Schematic overview of approaches to generate preclinical HNSCC models. **a** Patient-derived models are mainly generated from surgical tumor tissue. After mechanical and enzymatic dissociation, tumor cells are grown in vitro as 2D cell monolayers on plastic or 3D spheroid structures in extracellular matrix (ECM). For generation of patient-derived xenografts (PDX), tumor fragments are transplanted subcutaneously in immunocompromised mice. Classical patient-derived models are characterized by the absence of human immune and stromal cells. **b** Genetically engineered mouse models of oral squamous cell carcinoma can be generated by selective activation of oncogenes or inactivation of tumor suppressor genes (TSGs) in epithelial cells. **c** Delivery of 4-Nitroquinoline 1-oxide in the drinking water of mice over several weeks promotes oral cavity carcinogenesis at high incidence

Immortalized cell lines derived from HNSCC tumors represent a valuable tool for functional analysis of treatment resistance. Drug screening in monolayer cell cultures remain the common approach for identifying novel therapeutic agents. However, three-dimensional (3D) cultures which more closely represent tumor tissue architecture and cellular environment [7] might be superior for predicting drug efficacy in patients. Indeed, large variations in radiation and drug sensitivity have been shown in studies using 3D cell cultures, similar to those found with in vivo tumors. Even if 3D cultures are useful to study the interactions between different cell populations, they do not fully reproduce the complexity of HNSCC. Thus, development of novel therapies might ultimately require clinically relevant animal models of HNSCC that accurately represent the cellular and molecular changes associated with the initiation and progression of human cancer. In this respect, carcinogen-induced HNSCC models, transgenic animals and transplantable xenograft models have entered the field of HNSCC research. This review describes the mostly used preclinical models of HNSCC (schematically depicted in Fig. 1) and gives an overview of their strengths and limitations. We also discuss new approaches of personalized treatment selection based on these models.

### Ex vivo models

#### Immortalized HNSCC cell lines

Four decades ago, first protocols for ex vivo cultures of HNSCC cells have been reported [8, 9]. After resolving prior obstacles such as fibroblast overgrowth and dependence on feeder layers with these protocols, HNSCC cell lines were successfully established. Culture techniques have been further improved since then, and various HNSCC cell lines stably growing over numerous passages have been generated. A detailed description of all available HNSCC cell lines would be beyond the scope of this review. We would thus like to refer the reader to two previous review articles [10, 11]. Since immortalized HNSCC cell lines can be easily maintained and expanded, they have broadly been used to study genetic alterations and biological responses to chemical and genetic perturbations, to identify potential molecular targets, and to develop novel small-molecule and biological therapeutics [12, 13]. More recently, evidence has been provided that these cell lines can also be used for studying intratumoral heterogeneity and clonal evolution occurring under therapy pressure [14]. Data from such comprehensive molecular and functional studies in these models have been assembled in libraries like the Cancer Cell Line Encyclopedia (CCLE), representing a valuable repository of human cancer diversity [15, 16].

Although HNSCC cell lines grown in two-dimensional (2D) monolayer cultures continue to be important models in the search for new therapeutic approaches for this disease, they generally suffer from their inability to reflect the histological nature, three-dimensional (3D) architecture and structural and functional differences of the tumor in vivo. These limitations significantly influence the informative value of in vitro studies evaluating the efficacy of established and novel treatment modalities for HNSCC in monolayer cultures. Indeed, notable differences in sensitivity of 2D versus 3D cultures from HNSCC cell lines were reported for radiation [17] and drug treatment, e.g. with cisplatin [18], cetuximab [18, 19] and the mTOR inhibitor AZD8055 [19]. Comparative molecular analysis of cells growing in 2D versus 3D cultures provided possible explanations for lower sensitivity of cells in 3D cultures, like the expression and activation of genes associated with DNA repair [17], and increased expression levels of genes associated with epithelial-mesenchymal transition and stemness [18] under 3D conditions.

Genetic instability and the occurrence of clonal selection during in vitro *culture* [20] are further potential limitations of cancer cell lines, and can explain why findings involving cell lines are often difficult to reproduce. Indeed, comprehensive analysis of strains from the commonly used MCF7 breast and A549 lung cancer cell lines revealed extensive genomic variation across strains which was associated with variation in biologically meaningful cellular properties. Importantly, when the strains were tested against 321 anti-cancer compounds, considerably different drug responses were observed, with at least 75% of compounds strongly inhibiting some strains but being completely inactive in others. This study clearly underlines the urgent need for improved ex vivo models to support maximally reproducible cancer research.

#### Advanced ex vivo models of HNSCC

Köpf-Maier and colleagues were the first to establish a method which allowed human carcinoma cells from different histological entities including squamous cell carcinomas (SCCs) of the pharynx to reorganize in vitro to “organoid structures” [7]. They showed that these organoid cultures maintained the critical properties of the in vivo state, such as the 3D architecture, the growth of heterogeneous cell types from an individual carcinoma and the morphological differentiation under relatively simple experimental conditions [7]. In a subsequent study, the same group demonstrated that these organoid cultures can be used for drug testing, and that the response data obtained thereof were concordant with patients’ response to therapy [21]. The authors were the first to propose organoid cultures as personalized in vitro drug testing platform, allowing the prediction of individual chemosensitivity of carcinomas within few days [21].

Since then, techniques to grow tissues in vitro in 3D as organotypic structures have been refined. Protocols have been developed for establishing organoids from adult and embryonic stem cells which are able to self-organize into 3D structures that reflect the tissue of origin (for a review see Clevers, 2016 [22]). The first adult stem cell-derived organoid cultures were established from mouse intestinal stem cells that were placed in conditions mimicking the intestinal stem cell niche [23]. Conditional reprogramming induced by adding R-spondin-1, epidermal growth factor (EGF) and Noggin to the culture medium, and embedment of the cells in an extracellular matrix-providing basement membranes extract, has been shown to stimulate adult stem cells to self-renew, proliferate and form differentiated offspring, resembling the intestinal epithelium [23–25]. This technique, initially developed to study infected, inflammatory and neoplastic tissue from the human gastrointestinal tract, has not only been used for the establishment of organoid cultures from a variety of human normal tissue but also patient-derived tumor tissue. These studies have significantly enlarged and improved the set of available cancer models.

More recently, the early findings by Köpf-Maier and colleagues [21] of HNSCC organoid cultures being a suitable in vitro drug testing platform were confirmed by several independent studies. Though considerable differences in the success rates of establishing primary long-term growing organoid cultures from HNSCC patients were reported (30% [26] versus 65% [27]), all studies so far unanimously described that organoids retain many properties of the original tumor, including intratumoral heterogeneity [28], mutation profile and protein expression patterns [27, 29]. In addition, it was shown that organoids retained their tumorigenic potential upon xenotransplantation [27]. Responses to drug treatment in vivo were found to be similar to the IC50 calculated from organoids by drug sensitivity assays in vitro [26] Moreover, radiosensitivity data from organoid testing correlated with clinical response in patients [27]. Importantly, not only treatment-related effects in tumors but also unwanted therapy side effects in normal tissue can be studied in organoid models. For example, patient-derived salivary gland organoids have been used for dissecting the molecular basis of hyposalivation, a frequent severe side effect of radiation [30].

A further study identified primary 2D cell cultures from HNSCC patients’ tumors as additional valuable ex vivo HNSCC model [31]. Here, individualized large-scale screening of anti-cancer therapeutics reproducibly identified drugs displaying anti-tumor activity in matched patient-derived xenograft (PDX) models, thereby providing additional evidence that primary HNSCC cultures could be used to support therapeutic decision making in a routine clinical setting [31].

Organoid cultures of human normal, dysplastic, and malignant tongue tissues have also been used for reproducing the major steps of tongue tumorigenesis [32]. Histomorphometry, immunohistochemistry, and electron microscopy analyses in 3D co-cultures of tongue-derived primary keratinocytes and fibroblasts in collagen matrix showed that the stratified growth, cell proliferation, and differentiation were comparable between co-cultures and their respective native tissues, however, they largely differed in cultures grown without fibroblasts [32]. These results support previous studies showing an important role of cancer-associated fibroblasts in the pathogenesis of HNSCC [33]. These data together with broad evidence from the literature on tumor-promoting effects of the tumor microenvironment (TME) [33] strongly argue for future use of more advanced preclinical models comprising all major TME components. New protocols are nowadays available for generation of organoids containing beside stromal cells also the patient’s immune cells [34]. Thus, although organoid culture have limitations [35], such as the consumption of considerable time and resources and the incorporation of undefined extrinsic factors that may influence the outcome of experiments (Table 1), these cultures might represent suitable models to develop and optimize future treatment strategies including immune-oncology drugs.

**Table 1:** Advantages and limitations of preclinical HNSCC models

Preclinical model	Major advantages	Limitations	Pathogenesis modelling	Low-throughput drug screening	High throughput drug screening	Precision oncology^**a**^	Immune-oncology^**b**^
Immortalized cell lines	- low expenses - ease of maintenance - amenable to genetic manipulation	- chromosomal instability - low retention of genetic features	–	++	++	–	–
Primary 2D cultures	- high take rate - moderate expenses - amenable to genetic manipulation	- no resemblance of tumor architecture and cellular microenvironment	–	++	+	++	–
Organoids	- resemblance of tumor architecture - retention of genetic heterogeneity - reconstitution with stroma-immune components possible	- time and cost consuming - poorly validated HNSCC model - unknown effects of mouse-derived ECM components on cell behavior	++	++	±	++	++
Patient-derived xenografts	- retention of histological and genetic features of original tumor - tumor- (mouse-) stroma interactions	- time and cost consuming - reconstitution of immune system challenging	–	++	–	+	±
Carcinogen induced mouse models	- close resemblance of OC tumors - retention of genetic heterogeneity - immunocompetent model	- extended time until development of carcinomas - not all HNSCC sites can be modelled	++	+	–	–	±
Genetically engineered mouse models	- recapitulation of tumor initiation and progression - modeling of complex processes, e.g. tumor angiogenesis - immunocompetent model	- time and cost consuming - unpredictable frequency and latency of tumor formation - genetic alterations driving tumor formation rare in HNSCC	++	+	–	–	±

Potential applications were judged as suitable (++), possible (+), not very suitable (±) or unsuitable (−)

The suitability of the models for individual response prediction^a^ and development of immune-oncology drugs^b^ is given

*OC* oral cavity, *ECM* extracellular matrix

### Animal models

#### Carcinogen-induced animal models of oral cancer

Most human SCCs are known to be induced by chronic exposure to carcinogens. Initially, experimental approaches of inducing oral malignant tumors chemically always failed, because the oral mucosa was more resistant to the action of chemicals than skin. Finally, using 9, 10 dimethyl-1, 2, benzanthracene (DMBA) HNSCC could be successfully induced in hamster cheek pouch as an animal model [36, 37]. Resembling the scenario in patients, carcinogenesis of the mucosa occurred in four successive stages: hyperplasia, atypical hyperplasia, carcinoma in situ, and squamous cell carcinoma [37]. However, there were difficulties in distinguishing between changes of the epithelium caused by direct contact with the carcinogen versus true premalignant transformation because changes were transient and reversible in the DMBA-induced cheek tumors. In addition, DMBA-induced tumors did not possess many of the histological features of differentiated HNSCC and did not closely resemble early human lesions. More importantly, the tumor occurred in the hamster cheek pouch which represents an immunodeficient area absent in humans, so this model did not mimic human HNSCC very well. Although DMBA was subsequently widely employed in hamster and rat oral cancer models, it proved to be difficult to induce oral carcinoma with DMBA in mice [38]. 4-Nitroquinoline 1-oxide (4-NQO), a water-soluble quinolone derivative, was then introduced as potent inducer of oral tumors. Administration of 4-NQO with drinking water or its topical application resulted in multiple dysplastic, preneoplastic, and neoplastic lesions after long-term treatment in both rat and mouse models, and these lesions closely resembled human oral cavity neoplastic transformation. After several modification, the model was standardized by Tang et al. [39], who showed that delivery of 4-NQO in the drinking water of C57BL/6 mice for 16 weeks promotes oral cavity carcinogenesis at high incidence.

By recapitulating the sequence of events and the type of lesions seen during human carcinogenesis, the above-described carcinogen-induced animal models provide an excellent in vivo system for studying key driver events of oral carcinogenesis. These models have also been broadly used for the development of cancer chemoprevention strategies [40] whereas fewer studies have taken advantage of these animal models to evaluate the efficacy of drugs for treatment of established tumors. One major limitation as drug screening platform is the extended time needed to complete the evaluation of the effects of a test compound (Table 1). Most carcinogen-induced animal models of HNSCC require up to 40 weeks to develop full-fledged carcinomas, and even longer if metastasis is the study endpoint. In this context, a recent report from Wang and colleagues offers a potential shortcut by using 4NQO-derived cell line-induced tongue tumor xenografts as an alternative more expedient syngeneic mouse model [41].

The major advantage of the 4NQO-induced animal model is its suitability to study effects of carcinogenic and genetic factors in tumorigenesis especially in an immunocompetent environment. It thus provides a suitable platform for accelerating the development of immunotherapeutic regimens in HNSCC [41]. The model has also successfully been used to investigate the role of putative cancer stem cells in treatment resistance, recurrence, and metastasis. Its potential for developing novel therapeutic strategies targeting not only the proliferative tumor bulk but also the relatively quiescent subpopulation of cancer stem cells has been established [42].

#### Genetically engineered mouse models

While DNA damage by chemicals occurs randomly, according to the tumor evolution theory random acquisition of mutations across the genome is followed by selection of clones harboring genetic changes that facilitate cell survival and proliferation. Molecular profiling studies have identified several putative driver genes contributing to cancer development in HNSCC. However, these molecular studies did not provide direct evidence for causality or detailed insight into the biological mechanisms by which these genes drive tumor development. Although carcinogen-induced animal models can closely recapitulate the heterogeneous landscape of genomic alterations in human primary tumors [43], only a fraction of these mutations drive tumorigenesis by affecting oncogenes or tumor suppressor genes, but many mutations are passengers with no clear contribution to tumor development. These studies also do not reveal whether drivers are essential for tumor maintenance and may therefore be of limited use for designing effective therapeutic strategies. In contrast, preclinical model systems such as genetically engineered mouse models (GEMMs) provide an experimentally tractable approach in which the biological effects of specific mutations can be studied in detail in a controlled genetic background. In the next chapters, we describe key findings from previous studies based on GEMMs in HNSCC.

Few GEMMs associated with spontaneous HNSCC formation in the absence of chronic carcinogen exposure have been described so far (Table 2). A genetically engineered mouse model of oral cancer was first introduced by Schreiber and colleagues [44]. After crossing mice transgenic for the v-Ha-ras gene with transgenic mice that harbored E6/E7 of human papilloma virus (HPV)-16, the development of tumors at the mouth, ear and eye beginning at about 3 months of age was observed [44]. By 6 months, 100% of the bi-transgenic animals had developed oral tumors while the prevalence in either of the two single-transgenic groups was 0% [44]. The prerequisite of a second genetic hit for tumorigenesis was also reported for a transgenic model of K-*ras*^G12D^, in which a tamoxifen-inducible Cre recombinase under the control of the keratin-14 (K14) promoter was used for targeting the endogenous K-*ras* locus [45]. In the single-transgene model, only large papillomas in the oral cavity and hyperplasias in the tongue were observed after 1 month of tamoxifen treatment [45]. However, if mice were crossed with floxed p53 conditional knockout mice, 100% of the compound mice developed tongue carcinomas as early as 2 weeks after tamoxifen induction [45]. Beside expression of viral oncogenes E6/E7 and loss of *TP53*, homozygous deletion of the transcription factor krüppel-like-factor 4 (*KLF4*) [46] and heterozygous deletion of *SMAD4* [47] have been identified as second genetic hits which in concert with an oncogenic driver mutation promote oral tumor formation at high prevalence (Table 2).

**Table 2:** Transgenic models of HNSCC

Transgenic modification	Promotor	Anatomical site	Prevalence	Onset (months)	Reference
*Spontaneous models*					
E6/E7/mrasR12	K14	OC	100	3–4	[44]
KrasG12/TP53^del/del^	K14	T	100	< 1	[45]
KrasG12/KLF4^del/del^	K14	T	21	< 1	[46]
KrasG12/SMAD4^wt/del^	K14	OC	n.a.	3–4	[47]
SMAD4^del/del^	K14	OC	74	10	[47]
*4NQO-induced models*					
GRHL3^del/del^	K14	OC, L, P	40	3–4	[48]
PTEN^del/del^	K14	OC	100	1–2	[49]
miR-211	K14	T	n.a.	3	[50, 51]
miR-31	K14	T	80	3	[52]

*Abbreviations*: *K14* keratin 14, *OC* oral cavity, *T* tongue, *L* larynx, *P* pharynx, *n.a.* not available

Despite recapitulating HNSCC progression, the suitability of the above-described HNSCC models as platform for exploring novel molecular targeted treatment approaches remains somehow questionable, considering that the genetic alterations driving tumorigenesis in these animals are absent or only rarely found in HNSCC patients. Overall, mutations in *HRAS* and *KRAS* were detected in only 6 and 0.2% of HNSCC patients, and homozygous deletion of *KLF4* and *SMAD4* in 0 and 4% of cases, respectively. Moreover, cases harboring one of the compound tumor-prone genotypes of the GEMMs described above have not been identified in The Cancer Genome Atlas (TCGA) HNSCC cohort [53]. A GEMM of spontaneous HNSCC more closely resembling the molecular features of the human disease might be the single gene-knockout model of *SMAD4* in head and neck epithelia (HN-Smad4^del/del^) reported by Bornstein and colleagues [47]. Indeed, although homozygous deletion is rare, SMAD4 heterozygous loss is detected in 30–35% of primary HNSCCs [53, 54] associated with downregulation of Smad4 expression levels [53]. More recently, significant intratumoral heterogeneity of *SMAD4* loss in primary HNSCC tumors has been reported [54]. Interestingly, in ex vivo cultures derived from PDX, the cell subpopulation displaying heterozygous *SMAD4* loss by deletion or reduced expression outcompeted cells with wildtype *SMAD4* genotype from the parental tumor*,* suggesting a survival advantage of Smad4-deficient cells [54]. In further support of the suitability of this single-knockout GEMM, HNSCC from HN-Smad4^del/del^ mice exhibited increased genomic instability [47], which correlated with downregulated expression and function of genes encoding proteins in the Fanconi anemia/BRCA DNA repair pathway [47], also linked to HNSCC susceptibility in humans [55]. Moreover, both normal head and neck tissue and HNSCC from HN-Smad4^del/del^ mice exhibited severe inflammation which has also been linked to pathogenesis in humans [56] where oral bacteria and inflammatory mediators associated with periodontal disease may be co-factors in the initiation and promotion of oral SCC [57].

Since the original report in 2009, the HN-Smad4^del/del^ model has been used for analyzing in detail the molecular processes involved in HNSCC tumorigenesis. To our knowledge, it has not yet been exploited for the development of novel therapeutic strategies. This restraint might be explained by a median onset time of 40 weeks for tumor development in this model, a limitation similar to carcinogen-induced animal models of oral cancer (Table 2). The integration of carcinogen treatment to accelerate tumor formation in single-transgene GEMMs might thus represent an appropriate way to resolve this limitation, as already successfully demonstrated in studies of GEMMs harboring a deletion in a tumor suppressor gene (GRHL3 [48], PTEN [49]) or overexpressing oncogenic microRNAs [50–52] (Table 1).

#### Patient-derived xenograft models

The development and improvement of severely immune-deficient mouse strains has remarkably increased the availability of PDX models for cancer research. Successful establishment of HNSCC PDX models has been reported by several research groups [58–62]. In our own series, an overall engraftment rate of 48% was observed [60], however, engraftment rates seemed to largely vary between distinct patient subgroups [60, 61]. The limiting factors for engraftment have not yet been clearly identified. Site of implantation and mouse strains seem to influence the take rate. Moreover, pathological risk factors like tumor histology and HPV status are important determinants of PDX formation. In general, undifferentiated HPV-negative tumors displaying aggressive growth are more likely to engraft. Accordingly, the rate and kinetics of PDX engraftment have been associated with an unfavorable prognosis of patients [61–63]. Contrary to HPV-negative tumors, HPV-associated HNSCC tumors frequently fail to engraft. Since these tumors are growing at immune-associated sites such as the tonsil or base of tongue, their transplantation to immunodeficient mice lacking immunologic control of virally infected cells bears the risk of co-transferring Epstein-Barr virus (EBV) positive B-cells. As a result, uncontrolled B-cell proliferation and transformation to EBV+ lymphoma frequently occurs [60, 61]. Since the proliferation rate of these artificial lymphomas is much higher than tumor cell proliferation in transplanted tissue fragments of SCC, the original tumor transplants are frequently overgrown [60, 61]. Thus, histopathologic validation of PDX by a board-certified pathologist is essential to confirm the squamous cell carcinoma histology of the model.

The question of how well PDX resemble the primary patient tumor has been addressed by many groups. As shown for other tumor entities, established HNSCC models in mice display histopathologic features like the original patient tumor [59–62]. Comprehensive genetic analysis of primary tumors and derived PDX models by next generation sequencing revealed similar patterns and allelic frequencies of molecular aberrations [60, 62]. The correlation between mutational profiles of original tumors and derived models was significantly higher for PDX (*R* = 0.94) compared to cell lines (*R* = 0.51) [64]. Methylome analysis also showed high concordance between PDX and patient tumors. Indeed, an average of only 2.7% of the assayed CpG sites underwent major methylation changes as a result of transplanting tumors to mice [65]. Furthermore, gene expression studies showed the overall relatedness of parental tumors with their PDX, as confirmed by their clustering together in unsupervised hierarchical clustering analysis [59, 60]. In contrast to increasing evidence for genome and transcriptome profiles matching between PDX and primary HNSCCs, only few data exist for protein expression. A first preliminary analysis of PDX tissue using reverse-phase protein array (RPPA) revealed protein profiles comparable to the TCGA HNSCC protein expression data [66], suggestive of similarity between original tissue and derived model also at this level.

A key feature of PDX is the conservation of a stromal compartment. Even though human stroma is replaced by mouse stroma within the first passages, an integrated stroma remains which makes the evaluation of compounds targeting this compartment or crosstalk between stromal compartment and tumor cells possible. Further, tumors grown in mice build up their own tumor vasculature which offers the opportunity of evaluating the angiogenic network and interference with compounds targeting angiogenesis. After model establishment, tumors grown in mice can be harvested, vitally frozen and whenever needed thawed and re-transplanted to mice. Overall, PDX can be considered a suitable method for tumor tissue expansion, and a promising preclinical model system for mechanistic studies and the development of therapeutic strategies.

With the recent advent of immunotherapy in the treatment algorithm of many cancer types including HNSCC, the lack of a functional immune environment in PDX has become a major obstacle to overcome. Different strategies have been proposed to implement an immune system in immunodeficient mice. In the landmark study of Mosier and colleagues [67], it was shown that the injection of human peripheral mononuclear cells (PBMCs) resulted in the stable long-term reconstitution of a functional human immune system in mice with severe combined immunodeficiency (SCID). Thus, immunoproficient PDX models could be generated by transfer of patient’s PBMCs into the PDX-bearing mice. However, proper immune cell development and T-cell priming are lacking in this approach, resulting in the absence of certain lineages of human immune cells in mice [67]. More sophisticated immune reconstitution protocols subsequently developed are based on the transfer of human CD34+ stem cells into NSG mice, as well as implanting human fetal thymus and liver tissue under the kidney capsule of these mice [68, 69]. This approach resulted in the long-term engraftment and systemic reconstitution of a complete human immune system including multilineage human immune cells consisting of T-, B-, NK-, dendritic cells and macrophages [68, 69]. Unfortunately, this method is not feasible for a large number of PDX due to the complexity of the model. A more promising procedure has been proposed in melanoma where tumor-infiltrating T lymphocytes (TILs) isolated from the tumor tissue used for PDX generation were expanded in vitro by human interleukin 2 (IL2) before injection in tumor-bearing PDX mice [70].

#### The potential of PDX models to guide patient treatment

The value of PDX to guide individual patient treatment decision remains to be clarified. In general, patient-to-PDX correlations in different tumor entities comparing treatment responses between mice and patients have been done using retrospective data on clinical outcome. To our knowledge, no such comparisons at sufficiently large sample sizes have been performed in HNSCC. Obstacles for the worthiness of such approaches comprise drug dosage in mice which usually reflects the maximum tolerated dose, dose variability within different mouse strains and especially the definition of a clinically meaningful endpoint. In the clinical setting, tumor responses are determined by RECIST. In mice, a very heterogeneous set of possible endpoints has been used to determine the efficacy of single-drug treatments, including tumor regression expressed as relative growth inhibition, tumor volume in comparison to a control group, tumor growth inhibition and time to endpoint. Further general model limitations are the high cost of PDX establishment, varying engraftment rates and times from first transplantation to treatment screening results. So far, in our large collection of almost 80 HNSCC PDX models we have been unable to establish a predictive value of drug-specific tumor responses in the xenograft model. Nevertheless, several companies advertise PDX as a tool to predict treatment response. In 2016, Champions Oncology launched a feasibility trial (NCT02752932) to explore the predictive value of PDX. Unfortunately, no results have been published until now.

The main disadvantage of PDX is the prolonged time needed for model establishment and expansion compared to organoids, making their future use as individual drug screening platform in clinical routine less likely. In addition, re-constitution with patient-derived TME components which are missing in both models generated by current protocols should be achieved much easier in organoids than in xenograft mouse models. This will allow inclusion of anti-cancer therapies affecting the TME (e.g. everolimus, bevacizumab, anti-PD-1/PD-1 L antibodies) in future ex vivo screening approaches.

## Conclusions

Understanding the molecular mechanisms involved in the treatment resistance and progression of the disease, and elaborating new treatment strategies in preclinical models will significantly contribute to advance clinical management of HNSCC. While assays using HNSCC cell lines are essential for understanding HNSCC biology and drug development, they cannot serve as preclinical prediction model for the individual cancer patient. Organoid cultures resembling more closely the in vivo state may be more suitable in this respect, despite limiting factors such as higher costs and more elaborate maintenance required for these ex vivo cultures. GEMMs are useful in vivo models to interrogate the role of specific genes and genetic modifications in the pathogenesis of HNSCC. However, no single model described so far seems to be perfect for investigation of the pathogenesis and treatment of HNSCC. Though more studies are clearly needed to refine organoids and/or PDX as diagnostic tools for individual prediction of therapy response, it can be envisioned that the combination of molecular profiling of tumors together with drug testing in organoid models might significantly advance precision medicine in head and neck cancer, and improve the chances for patients to receive a treatment tailored to their tumor.

## Data Availability

Not applicable.

## Abbreviations

HNSCC: Head and neck squamous cell carcinoma
SCCs: Squamous cell carcinomas
DMBA: 9, 10 dimethyl-1, 2, benzanthracene
4-NQO: 4-Nitroquinoline 1-oxide
GEMMs: Genetically engineered mouse models
HPV: Human papilloma virus
TCGA: The Cancer Genome Atlas
PDX: Patient-derived xenograft
